# Global surgery for paediatric casualties in armed conflict

**DOI:** 10.1186/s13017-019-0275-9

**Published:** 2019-12-09

**Authors:** Frederike J. C. Haverkamp, Lisanne van Gennip, Måns Muhrbeck, Harald Veen, Andreas Wladis, Edward C. T. H. Tan

**Affiliations:** 1 Department of Surgery, Radboudumc, Geert Grooteplein Zuid, 9101, 618 Nijmegen, the Netherlands; 2 Department of Surgery, Linköping University, Norrköping, Sweden; 3 Department of Clinical and Experimental Medicine, Linköping University, Norrköping, Sweden; 4 Center for Disaster Medicine and Traumatology, Linköping University, Linköping, Sweden

**Keywords:** Paediatric trauma, Humanitarian aid, Global health

## Abstract

**Background:**

Understanding injury patterns specific for paediatric casualties of armed conflict is essential to facilitate preparations by organizations that provide medical care in conflict areas. The aim of this retrospective cohort study is to identify injury patterns and treatment requirements that are specific for paediatric patients in conflict zones.

**Methods:**

Characteristics of children (age < 15 years) treated in medical facilities supported by the International Committee of the Red Cross (ICRC) between 1988 and 2014 in Kabul, Kao-i-Dang, Lokichogio, Kandahar, Peshawar, Quetta and Goma were analysed; patient characteristics were compared between treatment facilities and with those of adult patients (age ≥ 15 years).

**Results:**

Of the patients listed in the database, 15% (5843/38,088) were aged < 15 years. The median age was 10 years (IQR 6–12); 75% (4406/5843) were male. Eighty-six percent (5012/5,843) of the admitted children underwent surgery, with a median of 2 surgeries per patient (IQR 1–3). When compared with adult patients, children were more frequently seen with fragment injuries, burns and mine injuries; they had injuries to multiple body regions more often and had higher in-hospital mortality rates.

**Conclusions:**

Children more often sustained injuries to multiple body regions and had higher in-hospital mortality than adults. These findings could have implications for how the ICRC and other organizations prepare personnel and structure logistics to meet the treatment needs of paediatric victims of armed conflicts**.**

## Introduction

The impact of war on children’s lives is extensive [1–4]. In conflict, the paediatric workload in military hospitals is 6% of all patients[5–10], and an even greater portion of paediatric patients (18%) is treated in humanitarian hospitals [11, 12]. Additionally, the percentage of paediatric patients is markedly higher among the surgical patient population in military hospitals (16%) [13, 14] as well as in humanitarian efforts (30%) [15–19], which again emphasizes that war-wounded children demand extensive care and resources [5, 8–10, 13, 20–24]. Additionally, younger age (≤ 8 years) has been independently associated with mortality in trauma patients admitted to combat support hospitals in Iraq and Afghanistan [9, 10, 22].

Given this context, it is of value to note that pre-deployment military medical training did not seem to fully meet the educational needs of their deployed personnel in the treatment of paediatric patients [25–27]. Our research group recently performed a survey among the medical personnel of the International Committee of the Red Cross (ICRC), a neutral and impartial humanitarian organization that provides assistance to victims of war. This survey revealed a need for additional training on the treatment of paediatric patients [28]. Furthermore, no referral centre for paediatric patients was available on most deployments, which demonstrates that these patients are largely dependent on ICRC’s medical facilities in conflict areas [28].

Consequently, the paediatric population in an armed conflict is at risk for many different reasons. It is therefore imperative to identify knowledge and skill gaps to improve treatment for paediatric patients. Detailed information on the injury patterns seen in these patients and their treatment needs is required. However, a recent report from Save the Children International on children in conflict zones between 1989 and 2016 revealed that there is a significant and worrisome gap in child-specific data [29].

The aim of this study is therefore to define patterns of injury and surgical treatment needs for paediatric patients in armed conflict zones. We analysed the demographic and epidemiological characteristics of paediatric patients who were admitted to and treated in eight different ICRC-supported medical facilities (see Table 1) and compared these data with those of adult patients and between treatment facilities. This information can be used for improving the preparation (i.e. medical training and logistics) of organizations providing medical care in conflict areas. The findings of this study could facilitate better preparation of deployed and local healthcare providers and result in more favourable treatment outcomes for young victims of armed conflict.

**Table 1:** Specifications per hospital

Hospital location	Geographical information	Period of data collection	Hospital opening date and closing date	Bed capacity	Facilities	Conflict details
Kao-I-Dang, Thailand	Southeast of Thailand, near Cambodian border	Jan. 1988 to Sept. 1992	1979–1993	1800	Paediatric wards, intensive feeding centre, gyn/ob wards, surgical wards, admission and emergency centre, tuberculosis centre, general medicine wards, two operating rooms, post-operative unit, laboratory, x-ray room	Cambodian civil war
Lopiding, Lokichogio, Kenya	Northern Kenya, near Sudanese border	Mar. 1988 to Mar. 2006	1987–2006	700	Two operating rooms, post-operative area, intensive care unit, multiple wards, physiotherapy unit, laboratory, pharmacy, orthopaedic workshop	Sudanese civil war
Kabul, Afghanistan	Northeast Afghanistan	Mar. 1990 to Jun. 1992	1989–1992	300	Two operating theatres with two and five tables, laboratory, X-ray service, blood bank	Afghan conflict
Quetta, Pakistan	Near Afghan border	Apr. 1990 to Aug. 1996	1983–1996	150	One operating theatre with three operation tables, laboratory, X-ray service, blood bank	Afghan conflict
Peshawar, Pakistan	Northern Pakistan, near Afghan border	Jun. 1990 to Apr. 1993 Feb. 2009 to May 2012	1981–1993 2009-2014	100 116	Three operating rooms, X-ray services, laboratories, physiotherapy services, intensive care unit	Afghan conflict
Mirwais, Kandahar, Afghanistan	Southern Afghanistan	May 1996 to Jun 1999	1996-still open	150	Three operating theatres, a blood bank, an X-ray service	Afghan conflict
Goma, Kivu, Democratic Republic of the Congo	Eastern Congo, along Rwanda’s border	Nov. 2012 to Oct. 2014	2012-still open	65	One operating theatre, physiotherapy, X-ray service, laboratory	Kivu conflict

*Gyn/ob* gynaecology/obstetrics

## Materials and methods

This study has been approved by the ICRC, Geneva, Switzerland. A retrospective review was performed of an ICRC database, which contained data from eight field hospitals in the following locations: Kabul, Kao-I-Dang, Lokichogio, Kandahar, Peshawar 1990–1993, Peshawar 2009–2012, Quetta and Goma (Table 1). In the field, these data were registered on paper. All cases were manually digitalized in an anonymous ICRC database using Microsoft Office Excel.

The following data were analysed for all paediatric patients aged < 15 years: sex, age, length of hospital stay (days), time to admission to hospital (hours), mortality, number of surgeries and blood transfusions, mechanism of injury and anatomical site of injury. These data were compared to those of adult patients (age ≥ 15 years) treated in the same treatment facilities.

The sample size was determined by data availability, as a fixed number of cases was available in the dataset. Missing data in this database is considered missing completely and at random; it was dealt with by restricting statistical analyses to individuals with complete data on the variables required for the analysis.

Data concerning paediatric patients were compared between the different treatment facilities and with adults using chi-square tests with post hoc *z* tests for categorical variables and (pairwise) Mann-Whitney *U* tests for continuous variables. A Bonferroni correction was used for multiple testing. Descriptive statistics are expressed in frequencies with percentages or medians with interquartile range (IQR). All statistics were calculated using SPSS statistical software (IBM SPSS Statistics for Windows, Version 25.0).

## Results

In total, data from 38,088 patients were recorded, of which 5,843 (15.3%) patients were aged < 15 years. Table 2 displays data on age, sex, length of hospital stay and mortality for paediatric patients. In Kabul, paediatric patients made up a greater part of the patient population (2185/6735; 32.4%) compared to other locations (*p* < 0.05); fewer paediatric patients were treated in Lokichogio (1110/13,406, 8.3%), Kao-I-Dang (97/1079; 9.0%) and Quetta (1043/7379; 14.1%; *p* < 0.05). Three quarters of the paediatric patients were male, and the male to female ratio was higher in Quetta (male 859/1034; 82.4%) and relatively lower in Goma (male 53/95; 55.8%) and Kao-i-Dang (male 61/97; 63%) compared to ratios from populations in other locations (*p* < 0.05); this ratio was also higher for adults (male 29,486/32,245; 91.4%) than for children (male 4406/5843; 75.4%; *p* < 0.05).

**Table 2:** Patient data per hospital location

	All patients									
	KAB	KAO	LOK	KAN	PES	QUE	PES2	GOM	Total	
Study period	2 years and 3 months	4 years and 8 months	18 years	3 years and 1 month	2 years and 10 months	6 years and 4 months	3 years and 3 months	1 year and 11 months	26 years and 9 months	
Total number of patients	6735	1079	13,406	1261	4579	7379	2960	689	38,088	
Patients < 15 years, *N* (%)	2185 (32.4%)	97 (9.0%)	1110 (8.3%)	186 (14.7%)	674 (14.7%)	1043 (14.1%)	453 (15.3%)	95 (13,8%)	5843 (15.3%)	
Patients ≥ 15 years, *N* (%)	4550 (67.6%)	982 (91.0%)	12,296 (91.7%)	1075 (85.2%)	3905 (85.3%)	6336 (85.9%)	2507 (84.7%)	594 (86,2%)	32,245 (84.7%)	
	Paediatrics									Adults
Hospital (*N*)	KAB (2185)	KAO (97)	LOK (1110)	KAN (186)	PES (674)	QUE (1043)	PES2 (453)	GOM (95)	Total (5843)	Total (32,245)
Median age, years (IQR)	10 (7–12)	8 (5–12)	10 (5–13)	8 (2–12)	10 (6–12)	10 (7–12)	10 (7–12)	9 (5–13)	10 (6–12)	26 (21–32)
Sex, *N* (%)										
Male	1622 (74.2%)	61 (63.0%)	820 (73.9%)	156 (83.0%)	504 (74.8%)	859 (82.4%)	333 (73.5%)	53 (55.8%)	4406 (75.4%)	29,486 (91.4%)
Female	561 (25.7%)	35 (36.0%)	290 (26.1%)	32 (17.0%)	169 (25.1%)	184 (17.6%)	120 (26.5%)	42 (44.2%)	1433 (24.5%)	2757 (8.6%)
Median LOS, days (IQR)^0^	8 (4–16)	10 (5–67)	33 (17–60)	11 (3–27)	10 (6–20)	19 (10–36)	15 (7–33)	21 (9–42)	13 (6–31)	17 (7–39)
Deaths, N (%)	119 (5.4%)	4 (4.0%)	22 (2.0%)	13 (7.0%)	26 (3.9%)	30 (2.9%)	24 (5.3%)	3 (3.2%)	241 (4.1%)	873 (2.7%)

Note that not all percentages in this table add up to 100%, which indicates the missing values

*LOS* length of stay, *IQR* interquartile range, *KAB* Kabul, *KAO* Kao-i-Dang, *LOK* Lokichogio, *KAN* Kandahar, *PES* Peshawar 1990–1993, *QUE* Quetta, *PES2* Peshawar 2009–2012, *GOM* Goma

Paediatric patients were hospitalized for shorter periods of time than adult patients (median 13 days for paediatric patients vs 17 days for adults; *p* < 0.05). The length of hospital stays for paediatric patients was significantly longer in Lokichogio (median 33 days; IQR 17–60) compared to all other hospitals (*p* < 0.05).

The time to admission (Table 3) could not be compared for patients treated in Goma because this variable was divided into different categories than those recorded by the other hospitals. Of all paediatric patients, 28.0% (1611/5,748) reached the treatment facility within 6 h after injury, whereas for adults, this percentage was 12.5% (3938/31,556). More often, it took adult patients over 72 h to reach the hospital (14,560/31,556; 46.1%; *p* < 0.05). Analysis by hospital showed that a greater proportion of paediatric patients took more than 72 h to reach the hospital in Lokichogio (834/1110; 75.1%) and Peshawar 2009–2012 (130/453; 28.7%; *p* < 0.05), whereas greater percentages of paediatric patients could reach the hospital within 6 h in Kao-i-Dang (61/97, 62.9%), Kabul (1200/2185; 54.9%) and Kandahar (69/186; 37.1%; *p* < 0.05). In Goma, most patients (50/95; 52.6%) could reach the treatment facility within 24 h.

**Table 3:** Time to admission

Hospital, *N* (%)	Paediatrics										Adults
	GOM (95)		KAB (2185)	KAO (97)	LOK (1110)	KAN (186)	PES (674)	QUE (1043)	PES2 (453)	Total (5748)	Total (31,556)
0–6 h 7–24 h 1–7 days 1–4 weeks > 1 month	23 (24.2%) 27 (28.4%) 17 (17.9%) 6 (6.3%) 10 (10.5%)	< 6 h < 24 h 24–72 h > 72 h	1200 (54.9%) 490 (22.4%) 184 (8.4%) 248 (11.4%)	61 (62.9%) 21 (21.6%) 10 (10.3%) 3 (3.1%)	30 (2.7%) 61 (5.5%) 142 (12.8%) 834 (75.1%)	69 (37.1%) 42 (22.6%) 19 (10.2%) 24 (12.9%)	139 (20.6%) 304 (45.1%) 118 (17.5%) 106 (15.7%)	69 (6.6%) 383 (36.7%) 269 (25.8%) 297 (28.5%)	43 (9.5%) 48 (10.6%) 75 (16.6%) 130 (28.7%)	1611 (28.0%) 1349 (23.5%) 817 (14.2%) 1642 (28.6%)	3938 (12.5%) 6582 (20.9%) 4925 (15.6%) 14,560 (46.1%)

Note that not all percentages in this table add up to 100%, which indicates the missing values

*KAB* Kabul, *KAO* Kao-i-Dang, *LOK* Lokichogio, *KAN* Kandahar, *PES* Peshawar 1990–1993, *QUE* Quetta, *PES2* Peshawar 2009–2012, *GOM* Goma

^a^Without Goma

Data on the mechanism and anatomical site of injury are displayed in Table 4. A comparison between children and adults demonstrates that paediatric patients were more often seen with fragment injuries (2112/5843; 36.1%), mine injuries (1197/5,843; 20.5%) and burns (195/5843; 3.3%), whereas adults were more frequently injured by gunshots (16,822/32,245; 52.2%; all with *p* < 0.05). Among paediatric patients, more fragment injuries were seen in Kabul (1246/2,185; 57.0%) and Kao-I-Dang (64/97; 66.0%) compared to other locations (both with *p* < 0.05); more gunshot wounds were seen in Lokichogio (653/1110; 58.8%) and Goma (59/95; 62.1%; *p* < 0.05). More mine injuries were seen in Quetta (367/1034; 35.5%), Peshawar 1990–1993 (258/674; 38.3%) and Kandahar (94/186; 50.5%; *p* < 0.05). However, in the period of 2009–2012, mine injuries were less frequently reported in Peshawar (36/453; 7.9%; *p* < 0.05).

**Table 4:** Mechanism and anatomical site of injury

Paediatrics										Adults
Hospital (*N*)	KAB (2185)	KAO (97)	LOK (1110)	KAN (186)	PES (674)	QUE (1043)	PES2 (453)	GOM (95)	Total (5843)	Total (32,245)
Mechanism of injury, *N* (%)										
Fragment	1246 (57.0%)	64 (67%)	154 (13.9%)	29 (15.6%)	260 (38.6%)	298 (28.6%)	47 (10.4%)	14 (14.7%)	2112 (36.1%)	7171 (22.2%)
Burns	96 (4.4%)	0 (0.0%)	16 (1.4%)	1 (0.5%)	11 (1.6%)	41 (3.9%)	23 (5.1%)	7 (7.4%)	195 (3.3%)	258 (0.8%)
Gunshot wounds	359 (16.4%)	5 (5.0%)	653 (58.8%)	48 (25.8%)	99 (14.7%)	224 (21.5%)	90 (19.9%)	59 (62.1%)	1537 (26.3%)	16,822 (52.2%)
Mines	388 (17.8%)	26 (27.0%)	28 (2.5%)	94 (50.5%)	258 (38.3%)	367 (35.2%)	36 (7.9%)	0 (0.0%)	1197 (20.5%)	4730 (14.7%)
Other	33 (1.5%)	1 (1.0%)	217 (19.5%)	3 (1.6%)	31 (4.6%)	95 (9.1%)	257 (56.7%)	15 (15.8%)	652 (11.2%)	2,496 (7.7%)
Anatomical site of injury, *N* (%)										
Head and neck	605 (27.7%)	26 (27%)	140 (12.6%)	40 (21.5%)	233 (34.6%)	272 (26.1%)	167 (36.9%)	8 (8.4%)	1491 (25.5%)	5199 (16.1%)
Thorax	244 (11.2%)	13 (13%)	88 (7.9%)	15 (8.1%)	81 (12.0%)	124 (11.9%)	68 (15.0%)	12 (12.6%)	645 (11.0%)	3606 (11.2%)
Abdomen	332 (15.2%)	19 (20%)	75 (6.8%)	32 (17.2%)	108 (16.0%)	158 (15.1%)	77 (17.0%)	14 (14.7%)	815 (13.9%)	2837 (8.8%)
Pelvis/buttocks	125 (5.7%)	3 (3%)	85 (7.7%)	11 (5.9%)	44 (6.5%)	56 (5.4%)	29 (6.4%)	4 (4.2%)	357 (6.1%)	2202 (6.8%)
Back and soft tissue of torso	72 (3.3%)	7 (7%)	39 (3.5%)	4 (2.2%)	30 (4.5%)	40 (3.8%)	27 (6.0%)	4 (4.2%)	223 (3.8%)	1315 (4.1%)
Upper limb	724 (33.1%)	31 (32%)	355 (32.0%)	63 (33.9%)	256 (38.0%)	387 (37.1%)	177 (39.1%)	12 (12.6%)	2005 (34.3%)	10,732 (33.3%)
Lower limb	962 (44.0%)	41 (42%)	489 (44.1%)	88 (47.3%)	301 (44.7%)	499 (47.8%)	244 (53.9%)	36 (37.9%)	2660 (45.5%)	16,284 (50.5%)

Note that not all percentages in this table add up to 100%, which indicates the missing values

*KAB* Kabul, *KAO* Kao-i-Dang, *LOK* Lokichogio, *KAN* Kandahar, *PES* Peshawar 1990–1993, *QUE* Quetta, *PES2* Peshawar 2009–2012, GOM Goma

The extremities were the most commonly injured body part in paediatric patients (3891/5843; 66.6%) as well as in adult patients (23,400/32,245; 72.6%), with the lower limbs (2660/5843; 45.5%) being injured more often than the upper limbs (2005/5843; 34.3%) in paediatric patients. Children more often sustained injuries to the head and neck (1491/5843; 25.5%; *p* < 0.05) and abdomen (815/5843; 13.9%; *p* < 0.05) compared to adults (5199/32,245; 16.1% and 2837/32,245; 8.8%, respectively). More limb injuries in paediatric patients were seen in Lokichogio (793/1110; 71.4%) and Peshawar 2009–2012 (340/453; 75.1%; *p* < 0.05). In Lokichogio, fewer injuries to the head and neck (140/1110; 12.6%), thorax (88/1110; 7.9%) and abdomen (75/1110; 6.8%) were reported (*p* < 0.05).

A total of 2,234 paediatric patients (38.2%) sustained injuries to multiple body regions; multi-trauma was reported less frequently in adult patients (10,060/32,245; 31.2%; *p* < 0.05). Fewer paediatric multi-trauma patients were seen in Lokichogio (237/1110; 21.4%; *p* < 0.05) compared to those seen in other hospitals, whereas more multi-trauma patients were seen in Quetta (459/1034; 44.4%) and Peshawar 2009–2012 (238/453; 52.5%; *p* < 0.05).

Mortality among paediatric patients (241/5843; 4.1%) was higher compared to adults (873/32,245; 2.7%; *p* < 0.05). For paediatric patients, mortality was highest in Kandahar (13/186; 7.0%) followed by Kabul (119/2185; 5.4%), and the latter was significantly different from all other locations (*p* < 0.05). Mortality was significantly lower in Lokichogio (22/1110; 2.0%; *p* < 0.05).

A total of 13,547 surgeries were performed on 5012 paediatric patients (85.8%) with a median of 2 surgeries per patient (IQR 1–3); 73.0% (3660/5012) of these patients had to undergo 2 or more surgeries. In adults, a total of 75,004 surgeries were performed on 26,853 patients (median 2, IQR 1–3), of which 77.4% (20,797/26,853) had to undergo 2 or more surgeries. Paediatric patients comprised 15.7% (5012/31,865) of the surgical patients; proportionally, paediatric patients were more frequently operated on (5012/5843; 85.8%) than adult patients (26,852/32,245; 83.3%; *p* < 0.05). Most paediatric patients (4820/5843; 82.5%) and adult patients (28,035/32,245; 87.0%) did not receive any blood transfusions.

## Discussion

This multicentre epidemiologic study provides extensive information on the epidemiology and demographics of the paediatric weapon-wounded patient population treated at eight different ICRC-supported medical treatment facilities. As opposed to many previous reports on weapon-wounded children, this study includes patients from multiple conflict zones over time.

This study shows that children make up a significant part of the patient population (15.3%) in ICRC-supported hospitals, have considerable surgery needs and often require multiple surgeries per individual. When compared with adult patients, children are more frequently seen with fragment injuries, burns and mine injuries. Sadly, our data reveal that children, more often than adults, are injured in multiple body regions and have higher in-hospital mortality rates.

The surgical workload for paediatric patients of 15.7% that was found in this study is closer to that in military hospitals (16%) [13, 14], than in other humanitarian efforts (30%) [15–19].This is expected since the ICRC mainly treats weapon-wounded patients. The unequal distribution of sex among children treated in hospitals in conflict areas, with the vast majority being male (overall male to female ratio of 4:1), is widely described in the literature [8, 11, 12, 16, 21, 24, 30, 31]. It has often been speculated that females are less likely to become injured in armed conflicts. However, over the years, females seem to be participating more actively in conflicts [30] and to be markedly affected by armed conflicts [11], which could be reflected in the decrease in the male/female ratio in the most recent time period of our study (Goma).

In the studied areas, paediatric patients often reached the hospital faster than adults. Generally, children are probably less likely to go outside on their own and will often have a supervising adult in close proximity who could take them to the hospital. With limited prehospital medical services and poor infrastructure in conflict areas, there might be more societal support to arrange transport to the hospital for injured children because of the emotional impact this has on witnesses.

Although paediatric patients had significantly shorter hospital stays than adult patients, the median length of hospital stay for children in this study (13 days IQR 6–31) was much longer than those reported in the literature concerning military hospitals (median ranging from 3 to 4 days) [7, 8, 10, 24]. Due to the difference in mandates between military hospitals and the humanitarian ICRC treatment facilities, children are likely to be transferred from a military hospital to a civilian medical facility after emergency care [32], whereas the ICRC treats patients until they are in no further need of in-hospital care.

The difference in the mechanism of injury seen between adults (gunshot wounds) and children (fragments, mines, burns) is also seen in casualties of the ongoing Syrian civil war [12] and could be because adults are more likely to be actively involved in the conflict. A previous study concerning ICRC data stated that during war, mines and fragmenting munitions are more likely than bullets to injure civilians [33].

Significantly more mine injuries were reported among paediatric casualties in Quetta and Peshawar (located in Pakistan close to the Afghan border) during 1990–1993 and Kandahar (Afghanistan) when compared with mine injuries from other locations. Our data from Peshawar 2009–2012 reveal a significant decline in mine injuries compared to those injuries from the early 1990s. We have reported on this previously, and other papers corroborate this finding [11, 32]. This decline in mine injuries was anticipated in Afghanistan, as large areas have been cleared of mines and unexploded ordnance since 1999; the same year, the Mine Ban Treaty came into force [34, 35]. A further decline in mine injuries should, hopefully, be expected from current conflicts in areas around participating states.

Very few paediatric patients had burns reported as the mechanism of injury, which is much less than reported in the literature [8, 10, 21, 24]. This underrepresentation is probably attributable to the high pre-hospital mortality rate of patients who sustained extensive burn injuries. Additionally, in the ICRC’s data, there could be an overlap between fragment, mine and burn injuries due to constraints in the classification of injury mechanisms. For example, a burn could be the most significant injury, but the injury could still be classified as fragments or mine if that was what caused the burn. The prevalence of burns could therefore be underestimated in this material.

Concerning the anatomical site of injury, our study results are in line with those of more recent epidemiological studies on paediatric injuries in Iraq and Afghanistan; the extremities were most frequently injured followed by the head and neck [12, 21, 24].

Regarding the different contexts studied, it is important to consider the possibility of pre-hospital patient selection, meaning that more severely injured children cannot gain access to medical care in time and die in the field. This is reflected by a lower percentage of paediatric patients with fewer critical injuries and a lower in-hospital mortality rate in Lokichogio, which was located further away from the conflict. Although in-hospital mortality rates were therefore generally low, paediatric patients showed a slightly higher mortality rate than adults, which could be explained by various reasons. Mainly, paediatric patients often reach the hospital faster than adults, which reduces the number of patients that deceases in the field. Furthermore, a higher mortality rate might indicate that more skills and expertise are required for this patient category; younger and more severely injured paediatric patients have shown to benefit from a higher level of paediatric expertise at the treatment facility [36]. However, the difference in mortality rate could just as well be caused by differences in body physiology and anatomy, mechanism of injury, and higher in-hospital prevalence of critical (head and neck) injuries in children. It has also been suggested that young children with conflict-related injuries may have an independent increased risk for death [22].

This study analysed differences in the epidemiology and demographics of paediatric patients between hospital locations and their contexts. The main findings may be mostly attributable to the differences in hospital location in relation to the conflict. However, any of the differences between hospital locations found in this study could also be attributed to differences in hospital logistics and the compliance of each individual employee with ICRC treatment protocols.

This study is not without limitations. Foremost, data acquisition can be impeded under field conditions in austere environments, and therefore, the accuracy of these data somewhat relies on the ability of ICRC medical personnel to keep a record of every patient and to thoroughly record every variable for each patient. This difficulty is reflected by the missing data for some variables and could have resulted in missing cases (patients) in our database. Additionally, the paper data were manually transferred into an electronic database, which poses the risk of coding mistakes.

Our data largely corroborate more recent data from military hospitals in conflict zones, but it would be preferable to compare our data with those of other humanitarian organizations located in conflict zones. The varying definitions of a child, ranging from < 15 years to < 19 years, also pose difficulties for comparisons with existing literature; hence, these comparisons should be interpreted with appropriate caution. An age of less than 15 years was used in this study as the definition of a child to ensure that a strictly paediatric population was studied. Additionally, this definition has been used in several previous studies [15, 23, 37, 38].

Although some of the patient data analysed in this study are somewhat dated, they provide a unique opportunity to study the evolution of paediatric injury epidemiology from conflicts over time.

The majority of our study subjects were victims of protracted conflicts in South Sudan (ICRC facility in Lokichogio) and Afghanistan (ICRC facilities in Kabul, Kandahar, Peshawar and Quetta), countries that face ongoing armed violence. During our study period, Afghanistan has been affected by rocket attacks and aerial bombings [39], strategies that are largely still used in modern conflicts [29]. Although the nature of these conflicts is subject to change, recent developments in modern warfare could increasingly affect civilians, including children. First, modern conflicts more often occur in densely populated urban areas [29]. Second, the nature of conflicts has shifted from confrontations between professional armies to one-sided violence, intrastate confrontations between the military and civilians or hostile groups of armed civilians [29]. Last, new technologies in weaponry design are increasing the distance between the user and the victim, which might cause the user to feel less responsible for his or her actions; this, together with the fact that these weapons can easily injure multiple people, could both contribute to the ongoing increase in the proportion of civilian casualties of armed conflict, as previously stated by Coupland et al. [33].

Due to the continued high prevalence of paediatric injuries in conflicts, the ICRC and other humanitarian organizations should deploy medical personnel who are skilled in treating paediatric trauma. This applies not only to surgeons but also to the whole scope of medical professions. The ideal situation would encompass deployment of highly skilled medical professionals with many years of experience in the (surgical) treatment of both paediatric and adult trauma patients. Considering the highly specialized medical professions nowadays, this is generally not feasible. Organizations could consider deployment of medical personnel primarily trained for paediatric trauma patients, but it is just as important to ensure greater participation of non-paediatrically trained personnel in basic courses or master classes focusing on paediatric casualties or to provide them with theoretical learning materials on this topic. Medical equipment on deployment should be suitable for paediatric populations, and deployed personnel should be provided the chance to become familiar with the equipment they will have at their disposal to treat paediatric patients during deployment.

## Conclusions

Children made up a significant part of the patient population resulting from war and armed conflict; they had considerable surgery needs, more often sustained injuries to multiple body regions and had higher in-hospital mortality rates than adults. These findings are important when training and preparing healthcare providers of all professions and specialties for work in conflict zones so that the providers can meet the needs of paediatric victims of armed conflict. Medical training and logistics should correspond to the actual treatment needs to optimize care and improve outcomes for the many children afflicted by war and armed violence.

## Data Availability

The datasets used and/or analysed during the current study are available from the corresponding author on reasonable request.

## Abbreviations

GOM: Goma
ICRC: International Committee of the Red Cross
IQR: Interquartile range
KAB: Kabul
KAN: Kandahar
KAO: Kao-I-Dang
LOK: Lokichogio
LOS: Length of (hospital) stay
PES: Peshawar
QUE: Quetta
